# Midline Incision of a Graft in Staged Hypospadias Repair–Feasible and Durable?

**DOI:** 10.3389/fped.2019.00060

**Published:** 2019-03-11

**Authors:** Ursula Tonnhofer, Manuela Hiess, Martin Metzelder, Doris Hebenstreit, Alexander Springer

**Affiliations:** ^1^Department of Pediatric Surgery, Medical University Vienna, Vienna, Austria; ^2^Department of Urology, Medical University Vienna, Vienna, Austria; ^3^Department of Urology, Medical University Innsbruck, Innsbruck, Austria

**Keywords:** hypospadias, two stage repair, midline incision, graft, buccal mucosa, preputial graft, complication

## Abstract

**Purpose:** In severe hypospadias staged repair is commonly used and it is regarded as feasible, safe, and durable. In this article we want to describe the results of a modification of the staged repair: a midline incision of the graft during the second stage.

**Materials and Methods:** This is a consecutive single team (2 surgeons) retrospective series. Between 2014 and 2017, 250 patients underwent hypospadias repair, among them 35 patients that had primary staged hypospadias surgery with completed first and second stage repair. 24 (68.6%) cases received a preputial skin graft and 11 (31.4%) buccal mucosa graft. Median age at first stage was 1.5 (0.5–22.1) years, mean time between first and second stage operation was 0.72 (0.4–1.76) years. Follow up rate was 100%, mean follow up period was 1.50 (0.4–3.8) years.

**Results:** The total complication rate was 22.9%. In buccal mucosa repair the complication rate was 36.4% and in preputial graft repair the complication rate was 16.7%, respectively. In 23 patients (65.7%) during second stage urethroplasty a midline incision was performed (8 glandular graft, 15 penile graft, 6 at level of urethral opening). Complication rate in non-incised urethroplasty was 8.3%, in incision at glandular level 37.5%, in incision at penile level 13.3% and in incision at urethral opening 16.7%, respectively.

**Conclusions:** Two stage repair is the method of choice in the correction of severe hypospadias. In selected cases a midline incision of the graft is feasible and can be applied if needed. Randomized studies will be needed to evaluate the true benefit of incising the graft.

## Introduction

Correction of severe hypospadias is a difficult task. Although there are many techniques available, today the two stage repair is the preferred method for many surgeons (1). In a first step, the penis is straightened and the urethral plate is replaced by a graft, i.e., preputial skin, other hairless skin, or buccal mucosa. In a second stage urethroplasty is performed. The staged repair is considered as feasible, safe and durable (2). However, in some cases second stage urethroplasty is challenging due to shrinkage of the graft, meatal stenosis, small glans, or other problems. We therefore in this article describe the results of a modification of the staged repair: a midline incision of the graft during the second stage.

## Methods

This is a consecutive single team (2 surgeons), single hospital retrospective series. Between 2014 and 2017, 250 patients underwent hypospadias repair. 48 patients had staged repair. Out of them we could identify 35 patients that had simple primary staged hypospadias surgery with completed first and second stage repair. 24 (68.6%) cases received a preputial skin graft and 11 (31.4%) received a buccal mucosa graft. Patient characteristics are shown in Table 1. Follow-up rate was 100%. Clinical follow up is scheduled for 3 months, 6 months, and then annually. In follow-up there is no routine urethral calibration, uroflow or objective cosmetic outcome assessment.

**Table 1:** Patient characteristics (*n* = 35).

Patient age at first stage	Median 1.5 (0.5–22.1) years
Patient age at second stage	Median 2.1 (1.1–23.1) years
Follow up	Mean 2.10 (0.8–4.4) years
Penile	7 (20%)
Penoscrotal	17 (48.6)
Perineal	11 (31.4%)
Preputial graft	24 (68.6%
Buccal/lingual mucosa	11 (31.4%)

### Surgical Technique

**Figure 1:**
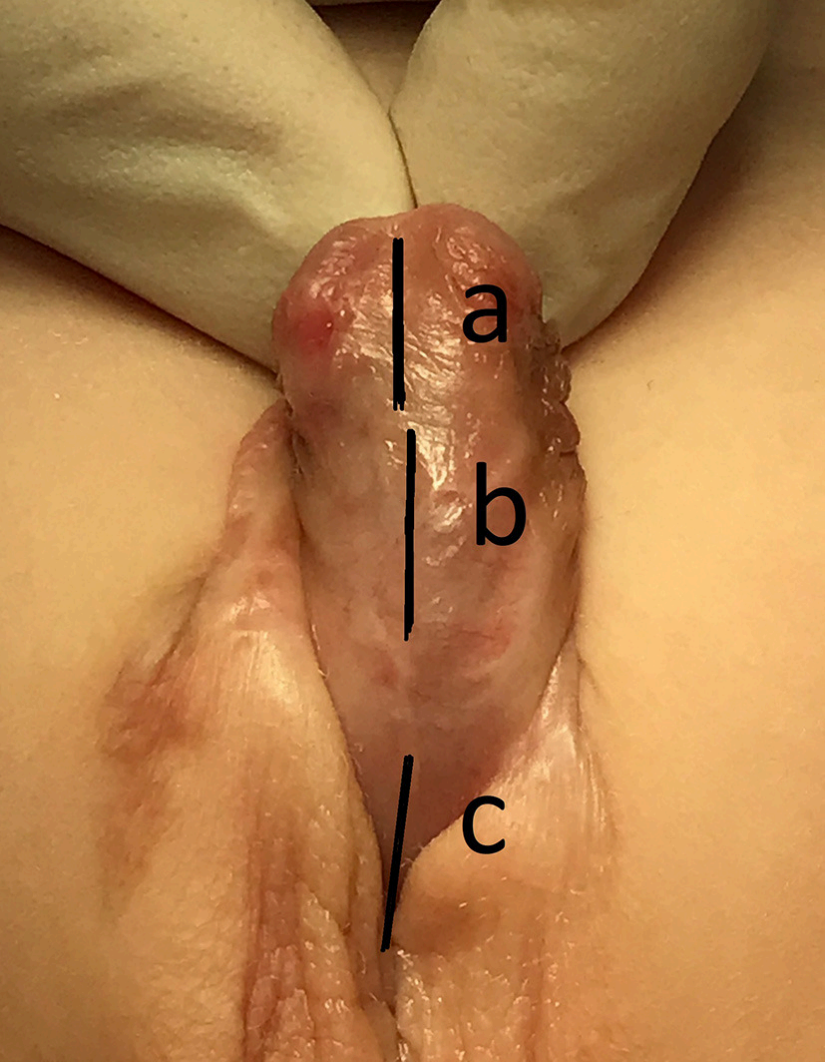
Ventral graft incision lines: **(A)** glandular graft/urethral plate when not wide enough to create a nice glandular urethra, **(B)** penile graft/urethral plate not wide enough and/or the scarred edges of the graft that do not allow wider incision lines **(C)** urethral opening when tight.

A first stage hypospadias repair is performed a described previously (3). Preputial grafts are preferred. Buccal mucosa is only used when there is a shortage of preputial tissues. Either a preputial graft or buccal mucosa is used as urethral replacement. Grafts are generously dimensioned as graft shrinkage has to be taken into account (particularly in buccal mucosa). During the second stage operation the margins of the graft/neourethra are defined and a 8 Fr. urethral stent is inserted. Using two toothed forceps the graft and glans are brought together and a midline incision of the previous graft is performed when (a) glans closure seems to be tight, (b) the graft is not wide enough to perform safe urethroplasty and/or too bulky to be nicely tubularized, or (c) the urethral opening is narrow and a 8 Fr. Catheter cannot be inserted easily (as shown in Figure 1). The procedure is depending on subjective judgment by the surgeon. There is a deep midline incision (level of corpora) as recommended in TIP. Second-stage urethroplasty is performed by tubularization using PDS 7.0 continuous subcuticular suture. One or two dartos layers are used to cover the urethroplasty using PDS 7.0 continuous sutures. Skin closure is performed with PDS 7.0 running mattress sutures. The penis is covered with Cavicare® and since 2016 with Alevyn® wound dressing. The stent is removed after 7 days.

## Results

In our institution a staged repair is performed when there is severe hypospadias [according to the SIU classification (4)]. The total complication rate was 17.1%. In 23 patients (65.7%) during second stage urethroplasty a midline incision was performed. Table 2 shows details and complications rates. All complications occurred during the first 3 months following second stage hypospadias repair and were recorded the 3 months standardized follow-up outpatient visit. All complications required surgery. There has been no objective scoring of the cosmetic outcome (PPPS, HOSE, or HOPE) but all parents seemed to appreciate satisfactory cosmetic outcomes.

**Table 2:** Graft incision and complication rate.

**Incision performed**	**Number (%)**	**Complications**	**Complication rate**
No incision	12 (34.3%)	1 breakdown	8.3%
Graft distal	8 (22.9%)	1 glandular breakdown, 1 fistula, 1 meatal stenosis	37.5%
Penile shaft	15 (42.9%)	1 fistula, 1 breakdown	13.3%
Urethral opening	6 (17.1%)	1 fistula	16.7%

## Discussion

Repair of severe hypospadias is difficult and there is a persistent high complication rate (5). Since the popularization of the two stage repair by Bracka in the late 1980ies it has been successfully applied to almost any form of hypospadias, from simple primary cases to difficult redo procedures (6, 7). Today, the two stage repair seems to be the most popular technique for proximal and difficult cases worldwide (1). Large series using different kinds of grafts (outer and inner prepuce, buccal mucosa, and others) have shown that the two stage repair is versatile and durable. Despite all the discussion about the “ideal” surgical technique for hypospadias repair, many other studies promote the advantages of the two-stage repair as save, reliable, and applicable to most of the cases (8–11). We standardized our clinical practice: In mild hypospadias a TIP procedure is performed. In severe hypospadias the two stage repair is favored. In our experience there has never been a failure in graft take. It has been stressed out that the graft should be harvested generously as graft shrinkage can be expected. Although the graft width should be as big as possible we were not able to identify the *ideal* width of the future neo-urethra during second stage urethroplasty. We screened the literature but objective parameters that allow to predict successful tubularization are still unclear (size of catheter, width of the incision lines). In our practice the margins of the neo-urethra are defined by the surgeon's experience. However, during second stage we sometimes are faced by the three following problems: (a) the glandular graft/urethral plate is not wide enough to create a nice glandular urethra (or the glandular groove is not deep enough), (b) the graft/urethral plate has shrunken and/or is not wide enough on the penile shaft and/or the scarred edges of the graft do not allow wider incision lines, (c) urethral opening is tight. Even in good graft take, buccal mucosa graft sometimes are bulky, thick, and not easily tubularizable. To address these issues a deep midline incision (like the TIP incision) has been introduced in our clinical practice.

Longitudinal incision of the tissue that should become the neo-urethra has been applied for many years (12). In 1994 Snodgrass described the tubularized incised technique (TIP) in the repair of distal hypospadias. Over the years, TIP repair has become the most popular technique for distal hypospadias repair worldwide (1, 13, 14). Meta-analysis and systematic reviews of large retrospective cohort studies show that there is enough evidence to recommend the TIP repair as versatile, highly standardized, and simple technique that provides favorable cosmetic and functional short and long-term results with a reasonable low complication rate (15–18). We conclude that the principle of the midline incision has been time proven. Experimental studies showed that longitudinal incision of the urethra does not induce scarring or fibrosis (19). On the other hand, internal urethrotomy (endoscopic midline incision of unhealthy tissues) in adult urology shows very dissappointing results with a high chance of recurring stricture or new scarring (20).

From hypospadias surgery point of view there are major differences between an untouched urethral plate and a well-taken preputial skin graft or buccal mucosa graft. Grafted tissues are thick and usually well-fixed to the underlying tissue (corpora cavernosa/tunica albuginea) (21). Often it is the availability that decides (for example in circumcised boys) or the need for a large amount of tissue. However, it is not well-described how preputial skin behaves as urethral replacement. Clinicially, the graft adopts well to a wet, urinous environment but due to the lack of long-term studies and limited availability of neo-urethral specimen for histology the fate of preputial grafts as urethra remains unclear and nebulous. Buccal mucosa, on the other hand, is well-adopted to moisture. In a systematic review and meta-analysis about urethral reconstruction in adults using buccal mucosa or penile skin grafts from 2012 it seemed that buccal mucosa was superior as urethral replacement but the evidence was low and there was bias in the length of follow-up (22). Moreover, the nature of the graft and the fate of these tissues have not been studied at all. For our purposes it would be highly interesting how the graft tissues react to deep longitudinal incision. Is there scarless healing and re-epithelialization like in a primary TIP repair? Or do we have to expect more scarring and difficult wound healing in a skin graft like in primary skin healing? There is no comparative study or experimental study available currently. When going through the literature we were surprised that incision of a graft at second stage urethroplasty has not been discribed in the literature before. Our indications were:

### Midline Incision of Glandular Graft

Although not widely reported, in severe hypospadias a coronal meatus may be cosmetically acceptable. However, most surgeons would at least try to have nice glandular urethra and glanduloplasty. A situation where a midline incision can be applied is when the glandular graft/urethral plate is not wide enough to create a nice glandular urethra. The graft might be bulky or the glans can have a shallow appearance or may be small in size. In our series in 22.9% of all cases a glandular incision was performed. No urethral stricture or meatal stenosis occurred. However, there was one glandular breakdown.

### Midline Incision of Penile Graft

In the majority of staged hypospadias repairs there is nice graft take and after 6 months or so the tissues are healthy, well-vascularized and subtle. Urethroplasty is performed easily and the outcome is favorable. However, in our series there are cases where the graft/urethral plate has contracted and primarily is not wide enough for safe urethroplasty. Moreover, we observed the scarred edges of the graft that do not allow wider incision lines. These things happen more in buccal mucosa than in preputial grafts. In buccal mucosa sometimes is bulky, thick, and not easily to be tubularized. Incision of the penile part of the graft is now routinely performed. In our series there had been 1 fistula, 1 breakdown, and 1 case of lichen sclerosus of the entire urethroplasty. Again, we did not encounter any stricture or problem of longitudinal scarring which might be expected following midline incision of the graft in the first place.

### Midline Incision of the Urethral Opening

To avoid meatal stenosis in the first stage the urethra is incised ventrally. However, in some cases there is scarring or fibrosis where the graft meets the original urethral tissue resulting in a tight urethral opening, sometimes so tight that an 8 Fr. catheter could not be inserted. This problem was addressed by a deep midline incision. In our series we could not identify any problem related to such an incision. There was no stricture recorded at the level proximal anastomosis. There was one distal fistula which can hardly be related to a very proximal incision.

Even in the largest series worldwide our modification of incising the graft was not used or not reported [Shukla et al. 700 cases (23), Bracka 600 cases (7), Obaidullah 1200 cases (11)]. However, from our experience it is very difficult to imagine that such an incision has not been performed by others in staged hypospadias repair. Although in the range reported in literature (24), complication rate in our series is high. Complication rate is particularly high in those patients where a midline incision had been performed. However, this is not a randomized study and probably there is bias. Most probably, midline incision of the graft was performed in more severe cases. Still we think that incision at second stage urethroplasty is a useful tool in selected cases (glandular urethra, primary meatal opening, and tight urethra or shrunken graft).

### Limitations

This is a single center retrospective short to mid-term study with a limited number of patients and longterm results are still pending. There is no control group and the technique of incising a graft has not been applied in a randomized setting but by surgeon's subjective judgment. Therefore, many facts remain unclear: for example: is the complication rate influenced by the incision or has the incision been applied in more difficult cases? Moreover, we do not routinely perform cystoscopy in hypospadias patients, therefore endoscopic findings following midline incision can not be reported.

## Conclusion

Two stage repair is the method of choice in the correction of severe hypospadias. In selected cases a midline incision of the graft is feasible and can be applied if needed. Randomized studies will be needed to evaluate the true benefit of incising the graft.

## Data Availability

The datasets generated for this study are available on request to the corresponding author.

## Ethics Statement

There is Medical University Vienna ethical committee approval for this study - number 2265/2017.

## Author Contributions

AS, UT, MH, MM, and DH contributed to study concept, design, and drafting of the manuscript. AS, UT, and DH contributed to acquisition of data. AS, UT, MH, and MM contributed to analysis and interpretation of data. AS contributed to critical revision of the manuscript for important intellectual content.

### Conflict of Interest Statement

The authors declare that the research was conducted in the absence of any commercial or financial relationships that could be construed as a potential conflict of interest.
